# Shared storybook reading with children at family risk of dyslexia

**DOI:** 10.1111/1467-9817.12375

**Published:** 2021-09-13

**Authors:** Lorna G. Hamilton, Marianna E. Hayiou‐Thomas, Margaret J. Snowling

**Affiliations:** ^1^ School of Education, Language and Psychology York St John University York UK; ^2^ Department of Psychology University of York York UK; ^3^ Department of Experimental Psychology and St John's College University of Oxford Oxford UK

**Keywords:** home literacy environment, shared reading, family risk of dyslexia, bioecological model, parent–child interaction

## Abstract

**Background:**

Shared storybook reading is an important context for language learning and often constitutes young children's first encounter with the printed word. The quality of early shared reading interactions is a known predictor of language and reading development, but few studies have examined these interactions in children at family risk of dyslexia.

**Methods:**

This exploratory study describes the quality of shared storybook reading between mothers and their 3‐ to 4‐year‐old children at family risk of dyslexia (FR; *n* = 18) in comparison with dyads with no known risk (no‐FR; *n* = 13). Mother–child interactions while sharing a familiar and an unfamiliar storybook were coded for type of extra‐textual talk (meaning‐related talk at the concrete and abstract levels; print‐related talk) and affective quality. Maternal and child language and literacy skills were considered as potential correlates of shared reading quality.

**Results:**

The linguistic and affective quality of shared reading was broadly comparable across FR and no‐FR dyads, particularly when sharing a book they knew well, with large within‐group variation. Mothers contributed more concrete meaning‐related talk when introducing an unfamiliar book to their children; children contributed more extra‐textual talk overall when sharing a familiar book. Maternal language, but not reading, skills were related to the linguistic quality of shared reading. The affective quality of reading interactions was rated more highly in dyads where mothers and children had stronger language skills.

**Conclusions:**

These results suggest that the quality of shared reading does not vary systematically as a function of children's risk of dyslexia but is related to maternal language skills. This finding needs to be replicated in a larger sample in order to better understand the risk and protective factors associated with dyslexia.

**Highlights:**

*What is already known about this topic*

- The quality of extra‐textual talk during shared reading between parents and preschoolers predicts later language and literacy outcomes in typically developing children.
- The affective quality of early shared reading predicts children's motivation to read independently in later childhood.
- Children at family risk of dyslexia are more likely than their peers with no family risk to have difficulty learning to read and may show weaknesses in oral language skills.

*What this paper adds*

- The linguistic and affective quality of shared reading between mothers and preschool children is broadly similar when children are at family risk of dyslexia compared with no family risk.
- The type and quantity of extra‐textual talk contributed by mothers and children appears to differ according to the familiarity of the storybook, but replication of the findings in a larger sample is required.
- The linguistic and affective quality of shared reading is related to maternal language skills.

*Implications for theory, policy or practice*

- Shared storybook reading offers rich language learning opportunities for children at family risk of dyslexia.
- Maternal language skills may be an important determinant of the interactional quality of shared reading.
- The linguistic and affective quality of shared reading is not clearly associated with maternal reading difficulties.

Shared storybook reading with adults provides a rich context for young children's language learning (Flack, Field & Horst, 2018). It is a particularly good setting for vocabulary development because children's storybooks typically contain a greater range of word types, and more abstract and sophisticated words, than everyday child‐directed speech (Dawson, Hsiao, Wei Ming Tan, Banerji & Nation, in press; Montag, Jones & Smith, 2015). Storybooks also contain more complex grammatical structures and narrative devices than everyday conversation and thus may facilitate language learning beyond the word level (Seidenberg & Macdonald, 2018). Indeed, both the frequency and quality of storybook reading interactions in the preschool years are associated with later language outcomes (Leseman & de Jong, 1998; Zucker, Cabell, Justice, Pentimonti & Kaderavek, 2013). Children at family risk of dyslexia (i.e., with one or more dyslexic first‐degree relatives) are approximately four times more likely than their peers to experience reading difficulties themselves (Snowling et al., 2019)  and there is evidence that strong oral language skills may be a protective factor in the literacy development of these children (Snowling & Melby‐Lervåg, 2016). The present study examines shared storybook reading interactions between preschool‐aged children at family risk of dyslexia and their mothers, as a potentially important early context for language learning before children start to learn to read formally at school.

Learning to read builds on the foundations of oral language (e.g., vocabulary knowledge) and emergent literacy skills (e.g., knowledge of print forms and functions) that children acquire through the preschool years (Whitehurst & Lonigan, 1998). Many of these foundational skills for literacy grow through interactions with adults and older children in the home environment, including during shared book reading (Demir‐Lira, Applebaum, Goldin‐Meadow & Levine, 2019). According to the bioecological model of development (Bronfenbrenner & Morris, 2006), genetic and environmental factors operate on children's development through ‘proximal processes’; these are regular reciprocal interactions between the developing child and the people, objects and symbols in her immediate environment. Grolig (2020) applies this model to shared reading in the home: triadic interactions between child, parent and book constitute a proximal process for children's language learning. The quality of the shared reading process is itself influenced by characteristics of the parent and child (e.g., ‘resource characteristics’ including language and cognitive skills) and of the book (e.g., psycholinguistic properties of the text and familiarity of the story).

During shared reading interactions, parent–child dyads often engage in discussion of aspects of the story beyond the printed words, referred to as extra‐textual talk. Adults' input during shared reading, in the form of extra‐textual comments, questions and feedback, acts as a scaffold to raise the level of children's narrative comprehension and extra‐textual contributions. Parental extra‐textual talk during shared reading interactions tends to be more lexically diverse and syntactically complex than their child‐directed speech in other communicative contexts and is predictive of children's later vocabulary knowledge, reading comprehension and motivation to read independently (Demir‐Lira et al., 2019). At the conceptual level, parental extra‐textual talk has been characterised according to its level of abstraction, or distance from immediately perceptible aspects of the text (e.g., Hammett, van Kleeck & Huberty, 2003; Tompkins, Bengochea, Nicol & Justice, 2017). Concrete extra‐textual talk can include labelling objects or characters and describing characteristics or actions, while abstract talk requires integration of information that is not immediately present in the text or pictures through inference generation, evaluation, explanation or prediction (Blank, Rose & Berlin, 1978). Parental extra‐textual talk at both levels is associated with children's developing language skills (Sparks & Reese, 2013; van Kleeck, Gillam, Hamilton & McGrath, 1997; Wasik, Hindman & Snell, 2016), and the level of abstraction that is optimal for language learning appears to depend on the prior language level of the child (Reese & Cox, 1999; Zucker, Justice, Piasta & Kaderavek, 2010). In an experimental study of children's word learning during shared reading, a scaffolded approach – characterised by low‐demand questions at first encounter of a new word, followed by higher‐demand questions later – facilitated children's performance on a definitions task and children with higher vocabulary knowledge at baseline learned more words overall (Blewitt, Rump, Shealy & Cook, 2009). Because parents typically adapt their extra‐textual talk according to their child's language competence (Barachetti & Lavelli, 2011; Yurovsky, Doyle & Frank, 2016), this represents a reciprocal relation in development, such that children's language skill affects the level of cognitive demand in parents' talk, which in turn provides a context for children to extend their language skills.

Children's active participation in shared reading is recognised as an important driver of language learning. There is evidence that responses to parental questioning about the story can enhance children's vocabulary learning (Sénéchal, 1997) and dialogic reading techniques tend to produce larger vocabulary gains than non‐interactive child participation in shared reading interventions (Mol, Bus, de Jong & Smeets, 2008; but see also Noble et al., 2019). In addition, shared reading interactions in which extra‐textual talk is highly contingent and reciprocal between parent and child are considered optimal for learning (Dickinson, Griffith, Golinkoff & Hirsh‐Pasek, 2012) with children's verbal contributions increasing with the repeated readings of a storybook (Fletcher & Finch, 2015; Schapira, Bergman Deitcher & Aram, 2020).

Shared storybook reading can in principle also provide a setting for learning about print forms and function. However, eye‐tracking studies suggest that very young children rarely fixate spontaneously on the text during shared reading, spending substantially more time looking at illustrations (e.g., Evans & Saint‐Aubin, 2005). Emergent literacy skills, such as knowledge of letter names and sounds, typically require direct instruction from adults, albeit this sometimes occurs in the context of shared storybook reading (Han & Neuharth‐Pritchett, 2015; Treiman, Decker, Robins, Ghosh & Rosales, 2018). Intervention studies in which parents or teachers are trained to introduce explicit references to print during shared storybook reading have shown positive effects on young children's early literacy skills (Justice & Ezell, 2000; Piasta, Justice, McGinty & Kaderavek, 2012).

Beyond linguistic features, a small number of studies focus on socio‐emotional aspects of shared storybook interactions. Shared reading with infants and young children provides an opportunity for parent–child bonding and predicts aspects of parenting, including warmth, sensitivity and reduced stress and harsh parenting in longitudinal studies (Canfield et al., 2020; Jimenez, Mendelsohn, Lin, Shelton & Reichman, 2019). Furthermore, research has linked the affective quality of shared reading to meaning‐related extra‐textual talk (Baker, Mackler, Sonnenschein & Serpell, 2001; Leseman & de Jong, 1998), to children's concurrent reading engagement (Bergin, 2001), and to later motivation to read independently (Baker et al., 2001; Sonnenschein & Munsterman, 2002). In contrast, in one study, affective quality was negatively related to parental attempts to engage children in decoding words (Baker et al., 2001).

Against this background, an important gap in the current evidence is the effects of shared book reading on children at high risk of problems with language and literacy, such as children at family risk of dyslexia (Snowling & Melby‐Lervåg, 2016). Dyslexia is highly heritable, and its aetiology is complex, involving the interaction of multiple genetic and environmental factors (Bishop, 2015). As well as sharing genes with their parents, children at family risk of dyslexia may encounter different early home literacy experiences – driven by parent and/or child characteristics (Scarborough, Dobrich & Hager, 1991), but little is known regarding such factors. The majority of the existing studies of the home literacy environment focus on quantitative measures, such as the amount of shared reading, the availability of print materials in the home and children's interest in reading. None of these measures are reported to differ systematically between children with and without family risk of dyslexia when socioeconomic status is equivalent across groups (Caglar‐Ryeng, Eklund & Nergård‐Nilssen, 2020; Laakso, Poikkeus, Eklund & Lyytinen, 2004; Torppa et al., 2007; van Bergen, de Jong, Maassen & van der Leij, 2014). In other studies, both family socioeconomic status and some aspects of the home literacy environment have been reported to differ according to family risk group (Dilnot, Hamilton, Maughan & Snowling, 2017; Esmaeeli, Lundetrae & Kyle, 2018; Hamilton, Hayiou‐Thomas, Hulme & Snowling, 2016). Only one study to our knowledge has examined the quality of shared reading interactions in such samples: Laakso, Poikkeus and Lyytinen (1999) observed mothers with and without dyslexia reading an unfamiliar book with their 14‐month‐old infants. Interactional behaviours did not differ between mothers with and without dyslexia and nor did children's interest in the book or participation in the interaction. In both groups, mothers engaged most often in describing pictures on the page, likely because they were reading to infants from an unfamiliar book. In a follow‐up study, Laakso et al. (2004) reported that children's interest in shared reading was predictive of oral language skills and letter knowledge only in the group of children not at family risk, although there was no group difference in children's interest at 14 or 24 months. This study, however, focused primarily on child behaviours during shared reading and did not consider mothers' interactional behaviours or socio‐emotional features of the interaction in relation to language and emergent literacy skills. Here, we build on these findings in an exploratory study of 3‐ to 4‐year‐old preschool children at family risk of dyslexia (FR), describing the linguistic and affective quality of shared reading with their mothers, in comparison with that in dyads with no family risk (no‐FR).

Drawing on findings within the bioecological framework, the main aim of the study was to describe the linguistic and affective characteristics of shared reading interactions between 3‐ and 4‐year‐old children at family risk of dyslexia and their mothers. We focused on dimensions of shared reading that have been related to positive child outcomes in the literature: (1) mothers' and children's extra‐textual talk relating to the meaning of the story at the concrete and abstract levels, (2) print‐related extra‐textual talk and (3) the affective quality of the interaction. Given that the characteristics of the child, parent and book are all expected to influence the quality of shared reading (Grolig, 2020), and the amount and quality of extra‐textual talk has been shown to vary with book familiarity (Schapira et al., 2020), we compared shared reading of a familiar with a new book. The study addressed the following research questions:

1. Does the quality of shared storybook reading differ between dyads in which children are at family risk of dyslexia compared with those with no known risk? While previous research does not provide strong evidence of variation in the home literacy environment according to FR status, it is possible that by the later preschool years, variation in children's oral language skills associated with family risk of dyslexia may affect the quality of shared storybook reading (Snowling & Melby‐Lervåg, 2016).
2. Does the quality of shared storybook reading differ when interacting with a familiar versus an unfamiliar book? On the basis of previous findings (Schapira et al., 2020), we expected that children would contribute more extra‐textual talk when sharing a book that they know well. We predicted that the type of extra‐textual talk contributed by mothers would also vary with book familiarity, with relatively more support for literal comprehension through concrete meaning‐related talk provided when reading an unfamiliar book, and more high‐level, abstract talk during the familiar book reading. We further expected that the affective quality of the interaction would be higher in the context of sharing a familiar book.
3. Is the quality of shared reading related to maternal and child language and literacy skills in FR and no‐FR dyads? Following the bioecological model, we expected to observe correlations between measures of interactional quality and both mothers' and children's objectively measured skills.

## Methods

### Design

Mother–child dyads were observed in two naturalistic shared storybook reading interactions: one with a familiar storybook and the other with an unfamiliar storybook. The order of reading of the two storybooks was counterbalanced between dyads. Observational data were coded for the quality of extra‐textual talk contributed by mothers and children and for the affective quality of the shared storybook reading interaction. Objective measures of mothers' and children's language and literacy skills were assessed as potential correlates of shared reading quality.

### Participants

Thirty‐one mother–child dyads took part in this study, a subsample of families participating in a larger prospective study of dyslexia (see Snowling et al., 2019, for details). A subsample of families from among the last children recruited to the overarching study, excluding children with clinically significant language impairment, was approached to request participation in the shared reading observations (Table 1). The resulting sample was equivalent across FR and no‐FR groups for child age and gender. In line with the larger study sample, maternal education was higher in the no‐FR group; however, family socioeconomic standing did not differ significantly across the groups. Children (16 girls and 15 boys) were aged between 3 years, 10 months and 4 years, 11 months at the time of observation.

**Table 1:** Sample characteristics: mothers and children

	FR (*n* = 18)	No‐FR (*n* = 13)	*t*	Cohen's *d*
Mothers				
Age (years)	36.50 (3.28)	36.54 (4.03)	0.03	0.01
Education level^1^	4 (2–6)	5 (1–6)		
Family socioeconomic status^2^	69.83 (23.82)	74.46 (26.42)	0.51	0.18
Maternal language composite^3^	−1.01 (1.94)	1.42 (2.20)	3.26	1.19
Vocabulary^4^	105.88 (11.36)	116.46 (14.82)	2.22	0.82
Grammar (max = 20)	9.44 (3.54)	14.08 (3.93)	3.44	1.25
Oral language^5^	−0.92 (1.05)	−0.22 (0.83)	2.00	0.74
Maternal reading composite^6^	88.33 (15.10)	101.12 (10.93)	2.60	0.95
Word fluency^4^	86.11 (13.20)	96.54 (11.47)	2.29	0.83
Nonword fluency^4^	90.56 (19.01)	105.69 (13.88)	2.44	0.89
Children				
Age (months)	53.22 (4.40)	50.92 (3.55)	1.55	0.57
Gender (% girls)	50%	54%		
Child language composite^6^	108.83 (10.47)	112.31 (8.63)	0.98	0.36
Vocabulary^4^	110.67 (12.04)	112.15 (9.01)	0.38	0.14
Grammar^4^	107.00 (12.89)	112.46 (10.53)	1.25	0.46
Emergent reading composite^6^	103.17 (12.04)	117.62 (15.33)	2.94	1.07
Letter sound knowledge^4^	107.78 (14.93)	120.54 (12.95)	2.48	0.90
Early word reading^4^	98.56 (12.71)	114.69 (20.22)	2.73	0.99

^1^ Median (range) on the scale: 1 = no formal qualifications; 2 = General Certificates of Secondary Education (compulsory U.K. school exams, aged 16) or equivalent; 3 = A levels (U.K. school leaving exams, aged 18) or equivalent; 4 = professional vocational qualification; 5 = undergraduate degree; and 6 = postgraduate degree.

^2^ Index of multiple deprivation postcode ranking (%).

^3^ Mean *z*‐score.

^4^ Standardised score.

^5^ *z*‐score.

^6^ Mean standardised score.

Of the participating children, 18 were classified as at family risk of dyslexia (FR), because at least one parent or full sibling was identified as dyslexic through formal diagnosis and/or meeting the research criteria (see Snowling et al., 2019, for full details). Of the participating mothers of FR children, 11 self‐reported as dyslexic. Mean standardised scores for language and literacy measures generally fell within the average range for mothers and children in the sample; however, mothers' language and reading skills were higher in the no‐FR group (*ds* = 1.19 and 0.95, respectively) and the children in the FR group showed weaker emergent reading skills than those not at risk (*d* = 1.07).

### Observation of shared storybook reading: procedure and coding

Each mother–child dyad selected a picture book that they knew well and enjoyed reading together. Variability in the choice of book was allowed in this reading interaction in order to maximise the naturalistic nature of the observation. Mothers were also asked to read a storybook provided by the researchers (*The Cow that Laid an Egg*; Cutbill, 2008) in order to minimise the variability introduced by book choice in this second interaction. This book was selected for its attractive illustrations, salience of text and narrative structure, judged likely to elicit extra‐textual discussion; it was established that none of the families had read this storybook previously.

All extra‐textual talk contributed by mothers and children, including exchanges occurring immediately before and after reading the book, was transcribed verbatim. Transcripts were divided into communication units (C‐units), which were defined as an utterance containing a verb and its arguments (Heilmann, Miller, Nockerts & Dunaway, 2010), and direct repetitions of utterances were removed. Utterances that did not directly relate to the storybook, such as maternal instruction (‘sit still’), feedback (‘good girl’) or comments unrelated to the reading interaction (e.g., ‘I'm hungry’) were also excluded from the dataset for the purposes of analysis. The total number of extra‐textual utterances calculated for mother and child in each interaction therefore comprised only utterances directly related to the storybook reading.

Shared storybook interactions were coded by research assistants, who were blind to the risk status of children, according to the following categories: (1) *concrete meaning‐related extra‐textual talk*: mothers' and children's utterances relating to the meaning of the story were classified as concrete or abstract. Following van Kleeck et al.'s (1997) coding scheme, concrete utterances included ‘matching perception’ (labelling pictures) and ‘selective analysis/integration of perception’ (describing characteristics of characters or objects observable in illustrations). (2) *Abstract meaning‐related extra‐textual talk* included ‘reordering/inferring about perception’ (inference generation, judgements about characters, objects or ideas) and ‘reasoning about perception’ (predictions and integrating general knowledge; for full details, see Appendix A). (3) *Print‐related extra‐textual talk*: included utterances containing direct references to print forms (e.g., mother eliciting letter names or sounds from children and children asking ‘what does that word say?’) and broader print concepts (e.g., page turning, references to author and references to the storybook reading procedures such as ‘Shall we start reading here?’). (4) *Affective quality* was assessed using the qualitative rating scheme developed by Sonnenschein and Munsterman (2002) (Table 2). Five dimensions of affective quality (reading expression, contact with child, reader appearance of involvement, child appearance of involvement and reader sensitivity to child's engagement) were each rated on a scale of 1 to 3, giving a maximum possible score of 15. Internal reliability for this scale was marginally acceptable (α = .68).

**Table 2:** Affective Quality Rating Scale (Sonnenschein & Munsterman, 2002)

Rating	Descriptor
*Reading expression*	
1	Monotonous, flat reading, little attention to punctuation.
2	Some tonal change, no imitation of voices, moderate expression.
3	Expressive multi‐tonal reading; imitation of character voices; expression suggests suspense, etc.
*Contact with child*	
1	No or very little contact.
2	Occasional or little contact, less than 50% of time.
3	Contact greater than 50% of time arm around child, sitting on lap.
*Reader appearance of involvement*	
1	Distracted behaviour, little smiling or laughing related to story, irrelevant questions.
2	Looks at book 25–75% of time, some appropriate smiling, laughing, asking questions.
3	Attends to story most of time, appears to enjoy story most of time, asks questions, smiling, laughing.
*Child appearance of involvement*	
1	Distracted behaviour, little smiling or laughing related to story, irrelevant questions.
2	Looks at book 25–75% of time, some appropriate smiling, laughing, asking questions.
3	Attends to story most of time, appears to enjoy story most of time, asking questions, smiling.
*Reader sensitivity to child's engagement*	
1	Displays none of behaviours listed in the succeeding text.
2	Displays 1 or 2 of the following behaviours: asks child if enjoying story, acknowledges child's feelings, periodic eye contact to gauge child's interest, attempts to recapture child's attention if waning.
3	Displays 3 or more of the listed behaviours.

Inter‐rater reliability for the coded observation variables, calculated using 30% of the data, was good (Cohen's κ = .73–.90).

### Measures: mother and child language and literacy skills

Mothers completed a battery of language and literacy tests approximately 1 year before the shared reading observation; child language and emergent literacy tests were administered as part of a larger battery conducted approximately concurrently with the observation, when children were 4 years old. Reliability coefficients reported in the succeeding text were calculated from the wider study sample (*N* = 260).

### Maternal language

#### Vocabulary knowledge

Mothers completed the Vocabulary subtest from the Wechsler Abbreviated Scale of Intelligence (Wechsler, 1999), requiring participants to provide definitions for words of decreasing frequency. Responses are scored on a 0–2 scale (α = .94).

#### Grammatical knowledge

Mothers completed a sentence‐combining task adapted from the Test of Adolescent and Adult Language (Hammill, Brown, Larsen & Wiederholt, 2007), giving a maximum score of 20 (α = .79).

#### Oral language ability

Mothers completed the Communication Checklist – Adult (Whitehouse & Bishop, 2009), a self‐report measure of language competence over multiple domains (phonology, vocabulary, syntax and pragmatics) (α = .91).

These three measures of oral language were moderately correlated in the current sample, *r*(31) = .42 to .44, *p*s *<* .05; a maternal language composite was computed using mean *z*‐scores to reduce the risk of outliers having an undue influence in the small sample and to increase the reliability of the measure.

### Maternal reading

Mothers completed the Word Reading and Phonological Decoding subtests of the Test of Word Reading Efficiency (Torgesen, Wagner & Rashotte, 1999; α = .88). Participants read as many words and nonwords respectively as they could in 45 seconds. The two subtests were strongly correlated in the current sample, *r*(31) = .72, *p* < .001; a mean standardised score composite was computed as the index of maternal reading.

### Child language

#### Vocabulary

In the *Receptive One‐Word Picture Vocabulary Test* (Brownell, 2000), the child selected the corresponding picture depicting a word they heard from a choice of four (α = .95).

#### Grammar

In the Clinical Evaluation of Language Fundamentals *Sentence Structure* subtest (CELF‐Preschool 2 UK, Wiig, Secord & Semel, 2006), the child heard a sentence and selected the matching picture from a choice of four (α = .88).

### Child emergent literacy

Children completed the *Letter Sound Knowledge* and *Early Word Reading* subtests from the York Assessment of Reading for Comprehension: Early Reading (Hulme et al., 2009). In the Letter Sound Knowledge task, children were asked to give the sounds for 32 single letters and digraphs (α = .95). In Early Word Reading, they read 30 simple regular and irregular words of increasing difficulty (α = .98).

The two measures of children's oral language were moderately correlated in the current sample, *r*(31) = .46, *p* < .01, as were the two measures of emergent literacy, *r*(31) = .66, *p* < .001. Composite scores for each construct were computed for use in the analyses.

## Results

### Characteristics of shared storybook reading by family risk status and book familiarity

**Figure 1:**
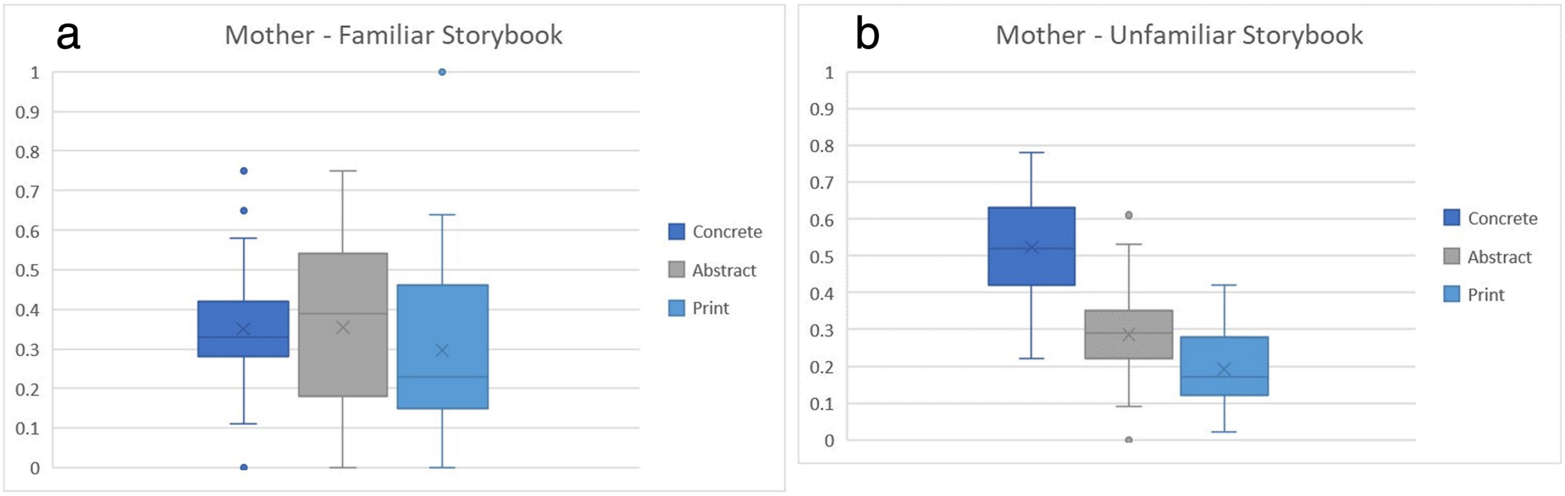
Proportion of maternal extra‐textual talk types across (a) familiar and (b) unfamiliar book readings.

**Figure 2:**
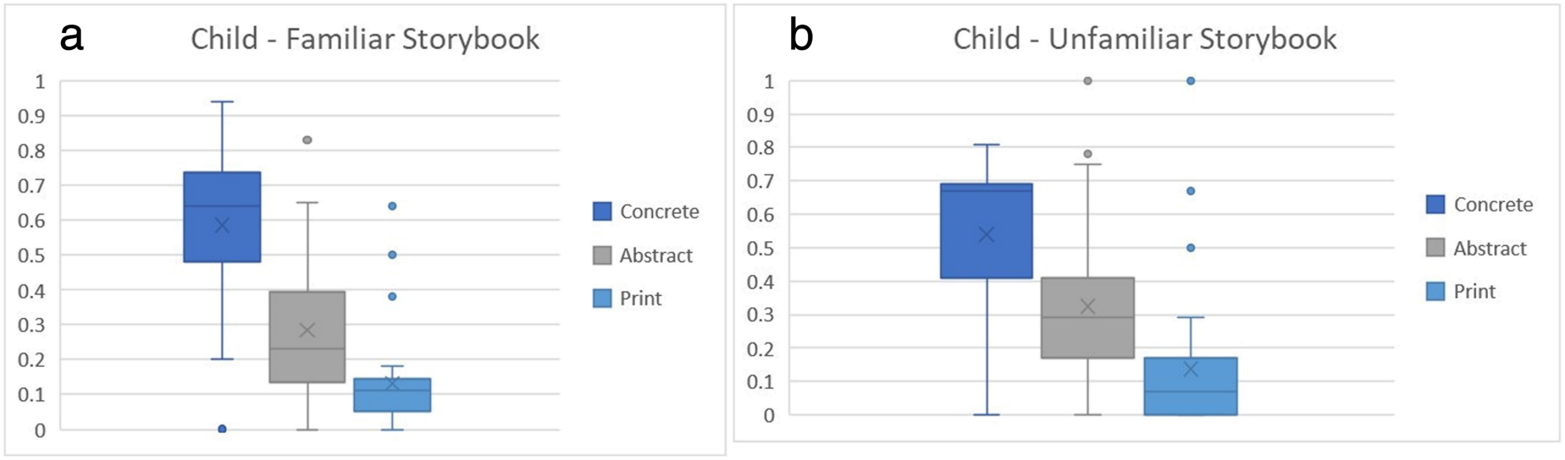
Proportion of child extra‐textual talk types across (a) familiar and (b) unfamiliar book readings.

Descriptive statistics for the indices of shared reading quality are reported in Table 3. The proportion scores for the different categories of extra‐textual talk across the two reading interactions are also presented in Figures 1 and 2.

**Table 3:** Shared reading observation: frequency counts of extra‐textual talk types and affective quality ratings according to risk status and book familiarity

	Familiar storybook		Unfamiliar storybook	
	FR	No‐FR	FR	No‐FR
	Mean (*SD*)	Mean (*SD*)	Mean (*SD*)	Mean (*SD*)
Mother total ETT	23.17 (22.26)	25.00 (17.69)	26.28 (12.91)	40.54 (28.16)
Concrete	9.33 (9.29)	8.38 (7.24)	13.78 (8.34)	22.08 (15.13)
Abstract	8.67 (9.49)	9.38 (7.86)	7.56 (5.08)	11.69 (8.85)
Print‐related	5.17 (5.11)	7.23 (5.89)	4.94 (4.11)	6.77 (6.03)
Child total ETT	21.67 (18.25)	21.85 (15.56)	11.89 (8.17)	18.38 (13.60)
Concrete	13.28 (12.56)	14.08 (12.86)	7.00 (6.31)	11.92 (9.32)
Abstract	6.22 (5.52)	4.85 (3.76)	3.56 (2.26)	5.08 (4.50)
Print‐related	2.17 (2.53)	2.92 (2.75)	1.33 (1.65)	1.38 (1.33)
Affective quality rating (/15)	11.59 (1.77)	11.77 (0.99)	11.05 (1.20)	11.51 (1.78)

*Notes*: ETT, extra‐textual talk; *SD*, standard deviation.

Given the small sample size and the wide variability in scores for all measures, extreme caution must be exercised in interpreting the data. However, a number of trends are worth noting. First, it can be seen from the table that, overall, mothers make more extra‐textual comments of all types than children, especially when reading an unfamiliar storybook. Further, the trends for children were generally in the opposite direction to those for mothers: they made fewer extra‐textual comments when the book was unfamiliar, in contrast to mothers who tended to make more comments in that setting. Another clear trend, observed for both types of book, was for mothers and children to make primarily meaning‐related comments and relatively few print‐related comments.

Comparing FR and no‐FR dyads, there was a trend for mothers in the no‐FR dyads to make more extra‐textual comments especially when sharing the unfamiliar storybook, and the increase was particularly observed for concrete meaning‐related talk, whereas that pattern was relatively less marked for mothers in the FR dyads. Further, the group differences between the FR and no‐FR children appear smaller than for the mothers.

Examining familiarity, there was a noticeable increase in the extra‐textual comments made by mothers in the no‐FR group when reading from an unfamiliar book, which was not nearly so marked in the FR group. This was coupled with a decrease in extra‐textual comments for the FR group of children (by about 50%) and a marginal decrease in the no‐FR group of children. In short, FR and no‐FR dyads appeared to behave more similarly to each other with a familiar book than when an unfamiliar book is introduced. Finally, reading interactions were rated as relatively high in affective quality across FR and no‐FR dyads, and there was no discernible difference in settings involving a familiar versus an unfamiliar book.

Correlations between the type of extra‐textual talk contributed by mothers and children across the two storybook reading episodes are reported in Table 4. Because all associations were comparable in strength when computed for the FR and no‐FR groups separately, data were pooled across the sample for these analyses. Most categories of extra‐textual talk were moderately to strongly inter‐correlated. However, when the total amount of maternal and child extra‐textual talk was partialled out to account for general differences between dyads in the propensity to converse during shared book reading (indicated in brackets in Table 4), the types of extra‐textual talk contributed by mothers and children showed more specific associations. Talk related to the meaning of the story at the abstract level was contingent between mother and child: there were moderate partial correlations between maternal and child abstract talk in both book readings, *r*_*s*_(28) = .41–.65. Mother and child talk was slightly less contingent at the concrete level, *r*_*s*_(28) = .25–.45, and in relation to print forms and functions, *r*_*s*_(28) = .31–.39.

**Table 4:** Non‐parametric correlations (Spearman's rho) between maternal and child extra‐textual talk types (frequency counts), pooled across FR and no‐FR dyads (*N* = 31)

	Mother concrete		Mother abstract		Mother print	
	Familiar	Unfamiliar	Familiar	Unfamiliar	Familiar	Unfamiliar
Child concrete						
Familiar	**.50 (.45)**	.51 (−.14)	.54 (−.24)	.67 (.16)	.47 (−.19)	.53 (.25)
Unfamiliar	.51 (.05)	**.73 (.25)**	.42 (.05)	.68 (−.38)	.26 (−.17)	.67 (.00)
Child abstract						
Familiar	.48 (−.10)	.01 (.16)	**.77 (.49)**	.31 (.01)	.41 (−.25)	.03 (−.37)
Unfamiliar	.27 (−.22)	.52 (−.12)	.63 (.59)	**.77 (.65)**	.24 (−.12)	.36 (−.44)
Child print						
Familiar	.38 (−.23)	.22 (.04)	.40 (−.29)	.39 (.04)	**.66 (.39)**	.27 (.04)
Unfamiliar	.01 (.03)	.05 (−.16)	−.05 (−.29)	.15 (−.07)	.20 (.27)	**.47 (.31)**

*Note*: Bracketed values represent partial non‐parametric correlations, controlling total maternal and child extra‐textual talk. Bold values show associations between mother‐child extra‐textual talk of the same type within the same shared reading episode.

### Associations between the quality of shared book reading and maternal and child skills

**Figure 3:**
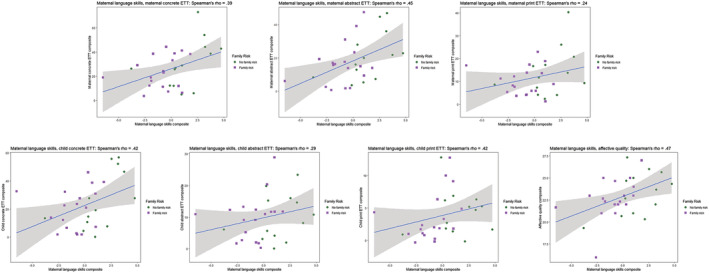
Scatter plots showing covariation between maternal language skills and indices of the shared reading quality; 95% confidence intervals around regression slope indicated by shaded area. ETT, extra‐textual talk.

**Figure A1:**
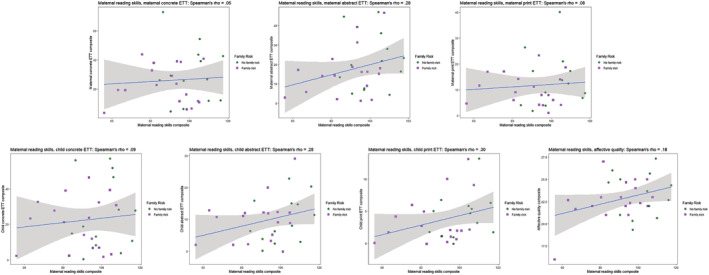
Scatter plots showing covariation between maternal reading skills and indices of the shared reading quality; 95% confidence intervals around regression slope indicated by shaded area. ETT, extra‐textual talk.

**Figure A2:**
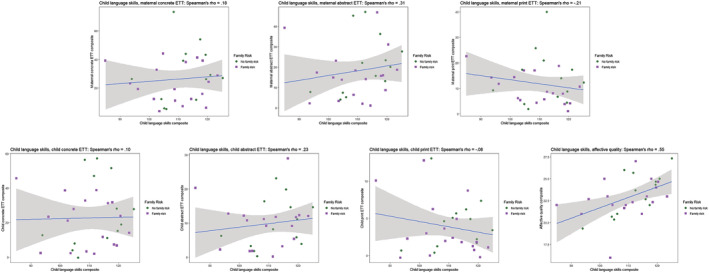
Scatter plots showing covariation between child language skills and indices of the shared reading quality; 95% confidence intervals around regression slope indicated by shaded area. ETT, extra‐textual talk.

**Figure A3:**
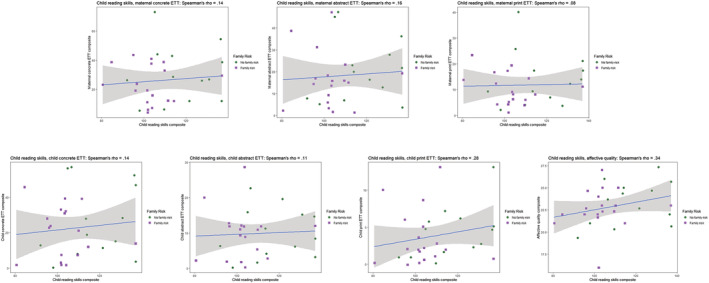
Scatter plots showing covariation between child emergent literacy and indices of the shared reading quality; 95% confidence intervals around regression slope indicated by shaded area. ETT, extra‐textual talk.

To assess the association between maternal and child language and literacy skills and the quality of shared storybook reading, we used visual inspection of scatter plots. Composite scores across familiar and unfamiliar settings were plotted with points coded to represent FR group; 95% confidence intervals of the regression line are indicated by the shaded area (Figures 3 and A1, A2, A3).

The plots show relatively wide dispersion around the regression line. Maternal language is positively associated with a number of indices of shared reading quality (Figure 3): maternal meaning‐related extra‐textual talk at the concrete level, *r*_*s*_(29) = .39, and abstract level, *r*_*s*_(29) = .45; children's concrete meaning‐related talk, *r*_*s*_(29) = .42, and affective quality, *r*_*s*_(29) = .47. The association between maternal language and children's print‐related talk, *r*_*s*_(29) = .42, should be interpreted with caution given the limited range in children's print‐related talk. In contrast to language skills, maternal literacy skills are uncorrelated, or weakly correlated, with the indices of shared storybook reading, *r*_*s*_(29) = .05–.28 (Figure A1).

Children's language and emergent literacy skills were largely unrelated to the linguistic quality of shared reading interactions (Figures A2 and A3), with the exception of a positive correlation between mothers' use of abstract meaning‐related extra‐textual talk and children's language skills, *r*_*s*_(29) = .31. The affective quality of storybook reading was also rated more highly in dyads where children had stronger language skills, *r*_*s*_(29) = .55.

## Discussion

This exploratory study describes qualitative aspects of shared storybook interactions between mothers and their preschool children, comparing dyads from families at risk of dyslexia with those carrying no family risk. Using naturalistic observations, it focused on the linguistic and affective quality of interactions when dyads shared a familiar and unfamiliar storybook.

At the group mean level, we observed trends but generally few differences between FR and no‐FR dyads in the amount or type of extra‐textual talk contributed by mothers and children. There was also no difference in the affective quality of shared reading interactions. Mean scores for all types of maternal and child extra‐textual talk were generally slightly lower in FR dyads, but there was also substantial within‐group variation in these measures. Overall, the verbal behaviour of FR and no‐FR dyads was more similar when sharing a familiar book in comparison with an unfamiliar book, when FR dyads tended to engage in less extra‐textual talk than no‐FR dyads. Given the small sample size, replication of the findings is important to avoid a Type II error. Nevertheless, it is notable that our findings are consistent with that of studies of home literacy practices according to FR status reporting minimal differences in the frequency or duration of shared reading or access to books in the home (Caglar‐Ryeng et al., 2020; Torppa et al., 2007; van Bergen et al., 2014), child interest in reading (Caglar‐Ryeng et al., 2020; Torppa et al., 2007) and interactional quality of shared reading with infants between the ages of 18 and 24 months (Laakso et al., 1999; Laakso et al., 2004). While the current study accords with this small body of literature on shared storybook reading in FR and no‐FR dyads with older preschool children, a limitation of all of the published studies is that they have recruited volunteer samples who may be especially motivated to read with their children.

Despite the trend‐level differences between FR and no‐FR dyads described earlier, there were also broad commonalities in the ways that mothers and children interacted with each other during shared reading of the two books. Consistent with Hammett et al. (2003), references to print forms and function were relatively infrequent during these shared storybook interactions in comparison with talk about the meaning of the story. Mothers in the current sample generally did not use shared storybook reading as an opportunity to teach their child about letter forms and print conventions. The dyads' verbal behaviour varied according to the familiarity of the book being read broadly as predicted. In line with the Vygotskian principle of scaffolding (Vygotsky, 1978), mothers talked more about the meaning of the story at the concrete level when reading an unfamiliar book to their child, compared with a familiar book. At first reading of a new book, mothers supported their child's basic understanding by labelling pictures, describing objects, characters and actions, and asking literal comprehension questions. This type of extra‐textual talk reduced when reading books that children knew well. Familiarity of the storybook also impacted children's verbal behaviour during shared reading; children contributed more extra‐textual talk overall when sharing a familiar storybook and were more likely to be quiet (it is assumed in order to listen) at the first reading of an unfamiliar book, in line with previous studies (Fletcher & Finch, 2015; Schapira et al., 2020). However, the affective quality of the interactions was rated as equally warm and responsive across readings of the familiar and unfamiliar books. These differences in interactional quality across the familiar and unfamiliar storybooks underline the triadic nature of shared storybook reading (Grolig, 2020).

To examine the resource characteristics expected to influence the proximal process of shared reading within the bioecological model, we also asked whether maternal and/or child language and literacy was related to the quality reading interaction observed. We found that mothers with better language skills talked more about the story in general and particularly at the abstract level, and their children talked more at the concrete level. Affective quality was also rated more highly in dyads where mothers had stronger language skills. These findings chime with those of Puglisi, Hulme, Hamilton and Snowling (2017) who reported that storybook reading mediates the impact of maternal language on children's language and emergent literacy skills, while direct instruction about print has an independent influence. They went on to show that maternal language skill predicted a latent variable describing children's storybook exposure in the home at age 4 (frequency, availability and checklist measures). Furthermore, while storybook exposure predicted child language and emergent literacy at age 5, including maternal skills in a longitudinal model meant that storybook exposure was no longer a significant predictor. While any causal interpretation of these preliminary findings that maternal language skills are related to measures of shared storybook reading quality is unwarranted, the proximal processes described in the current study (e.g., contingent extra‐textual talk between mother and child, particularly at the abstract level) may be one way in which gene–environment correlation mechanisms operate on children's language development.

In contrast to our findings on language, our data did not suggest that maternal reading skills were robustly associated with the quality of reading interactions. While reading ability is a continuous trait, and there is no clear‐cut distinction between ‘dyslexia’ and ‘not‐dyslexia’, the current sample included 11 mothers who self‐reported as dyslexic. Our data do not provide any reason to suppose that these mothers engaged in less linguistically rich or socio‐emotionally satisfying shared reading interactions than mothers without reading problems nor did we find clear relationships between child characteristics and linguistic features of extra‐textual talk. However, the affective quality of reading interactions was positively related to the language skills of both mother and child.

In summary, book sharing between parents and their young children is an important process for early language development. Within a bioecological framework, such proximal processes are conceptualised as the mechanism through which bidirectional transformations between the child and her environment occur (Bronfenbrenner & Morris, 2006; Grolig, 2020). This approach to development also proposes that proximal processes themselves vary as a function of characteristics of the developing child and the people and objects in her environment. The current study addressed three main questions: (1) Does the quality of shared storybook reading differ between dyads in which children are at family risk of dyslexia compared with those with no known risk or (2) when interacting with a familiar versus an unfamiliar book, and (3) is the quality of shared reading related to maternal and child language and literacy skills in FR and no‐FR dyads? Our data do not suggest that family risk of dyslexia affects the quality of interactions between mothers and their children when reading familiar or unfamiliar books; shared reading quality does, however, appear to be related to maternal language skills. The familiarity of the book being read also appears to influence the linguistic interaction of mother and child. Ideally, the findings need replication in a natural setting; they cannot be generalised to everyday life situations in which it remains possible that there are fewer episodes of literacy interaction in homes where one or other parent is dyslexic.

The present study offers proof in principle that it is practicable to rate different aspects of the linguistic and affective quality of reading interactions between 3‐ and 4‐year‐old children at family risk of dyslexia and their mothers, some of whom are themselves dyslexic. However, it has several important limitations, the most notable being the lack of statistical power given the small sample of mother–child dyads observed. The data are also cross‐sectional, which limits their utility. The study requires replication on a large sample, preferably involving longitudinal follow‐up, in order to elucidate causal mechanisms underpinning children's language learning during shared reading. The study was exploratory with the modest aim of characterising features of the shared reading interaction that may be important for language learning in children at family risk of dyslexia and expanding on our findings drawn from the same overarching study that quantitative measures of the home literacy environment are important predictors of later child language (Hamilton et al., 2016).

## Conclusions

This study adds to the small literature on parent–child shared reading in families with a history of dyslexia. Both FR and no‐FR dyads talked about the meaning of the story more than about features of the print. Children engaged in more extra‐textual talk when sharing a familiar book; mothers scaffolded children's understanding of an unfamiliar book by increasing extra‐textual talk at the concrete level. Maternal oral language, but not literacy, skills related to the linguistic quality of shared reading, while children's language and emergent literacy skills did not systematically relate to the amount or type of extra‐textual talk. The affective quality of shared reading was rated highly across FR and no‐FR dyads and was related to maternal and child language skills. These findings provide an illustration of proximal processes likely to be involved in language learning in children at family risk of dyslexia.

## Funding information

This study was funded by Wellcome Trust Grant R10611.

## Data Availability

The data that support the findings of this study are available from the corresponding author upon reasonable request.

## Appendix

**Table A1:** Levels of abstraction in meaning‐related extra‐textual utterances

Concrete		Abstract	
Level 1: matching perception	Level 2: selective analysis/integration of perception	Level 3: reordering/inferring about perception	Level 4: reasoning about perception
**Label:** name an object or person (or stated as question, e.g., ‘What's that?’) including negative label (‘It's not an X’). **Locate**: describe the location of an object or character; ask a question regarding location. **Notice:** direct attention to a pictured object. **Rote counting**	**Describe characteristics**: focus on perceptual properties (size, shape and colour) or parts of objects or characters. This includes colours or numbers if there is a referent. Specify the type of object, quantity of something or possession. **Describe/notice scene:** describe or notice actions that are immediately perceptual in text or pictures. **Complete cloze task:** mother leaves pause to allow child to complete sentence, or child completes sentence.	**Infer:** based on pictures/text and not explicitly stated/shown in pictures. **Recall information:** focus on prior information presented in book during current or previous reading; summarise/synthesise information from series of pictures. **Judgement/evaluation:** (about characters, objects or ideas) included non‐perceptual qualities and internal states (sad and hungry); sometimes introduced by epistemic verb (I think); judgements (beautiful and funny); providing point of view; and interpretation of what character is thinking or feeling. **Identify similarities:** compare or contrast between things in book.	**Predict:** offer or request what will happen next in the story (in unfamiliar storybook only, otherwise it is Level 3 recall). **Factual knowledge/information:** provide general information that is not directly given in the story. Includes defining word meanings or distinguishing between fantasy and reality. **Explain:** going beyond story or actions to provide an explanation, often indicated with words like ‘because’, ‘so that’ and ‘since’ or by asking ‘why’ questions.

*Note*: Based on the Hammett et al. (2003) adaptation of Blank et al. (1978).
